# Effect of pulse pressure on borehole stability during shear swirling flow vibration cementing

**DOI:** 10.1371/journal.pone.0187279

**Published:** 2017-11-16

**Authors:** Zhihua Cui, Chi Ai, Lei Lv, Fangxian Yin

**Affiliations:** 1 School of Petroleum and Environmental Engineering, Yan′an University, Yan′an, Shaanxi Province, China; 2 Key Laboratory of Education Ministry for Enhanced Oil Recovery, Northeast Petroleum University, Daqing, Heilongjiang Province, China; 3 School of Mechanical and Materials Engineering, North China University of Technology, Beijing, China; China University of Mining and Technology, CHINA

## Abstract

The shear swirling flow vibration cementing (SSFVC) technique rotates the downhole eccentric cascade by circulating cementing fluid. It makes the casing eccentrically revolve at high speed around the borehole axis. It produces strong agitation action to the annulus fluid, makes it in the state of shear turbulent flow, and results in the formation of pulse pressure which affects the surrounding rock stress. This study was focused on 1) the calculation of the pulse pressure in an annular turbulent flow field based on the finite volume method, and 2) the analysis of the effect of pulse pressure on borehole stability. On the upside, the pulse pressure is conducive to enhancing the liquidity of the annulus fluid, reducing the fluid gel strength, and preventing the formation of fluid from channeling. But greater pulse pressure may cause lost circulation and even formation fracturing. Therefore, in order to ensure smooth cementing during SSFVC, the effect of pulse pressure should be considered when cementing design.

## Introduction

**Fig 1:**
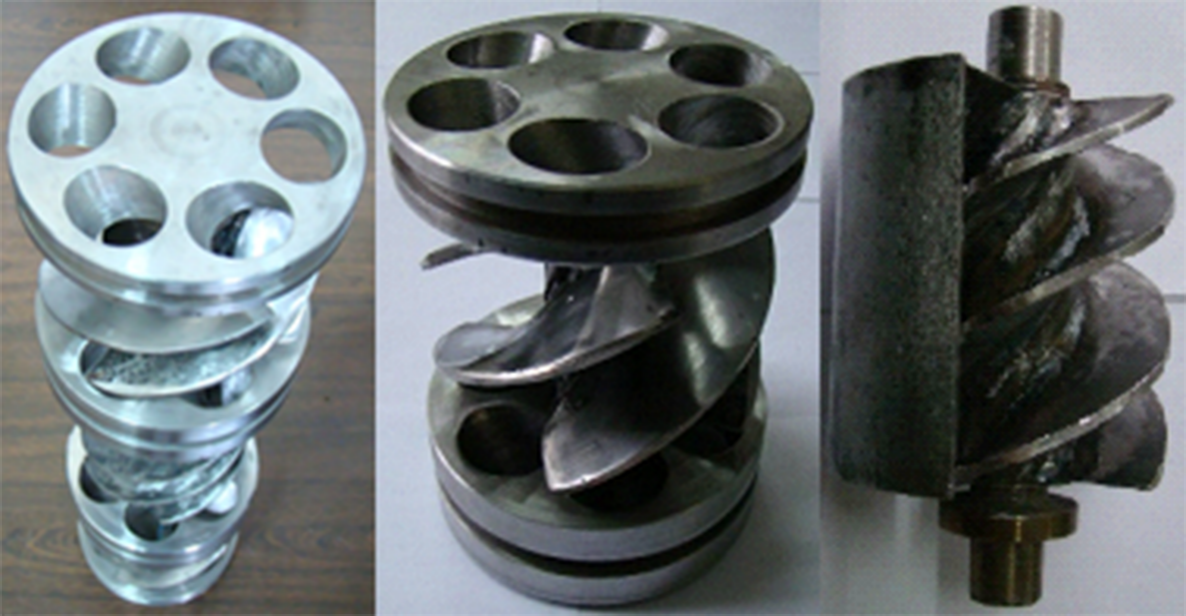
The downhole eccentric cascade.

**Fig 2:**
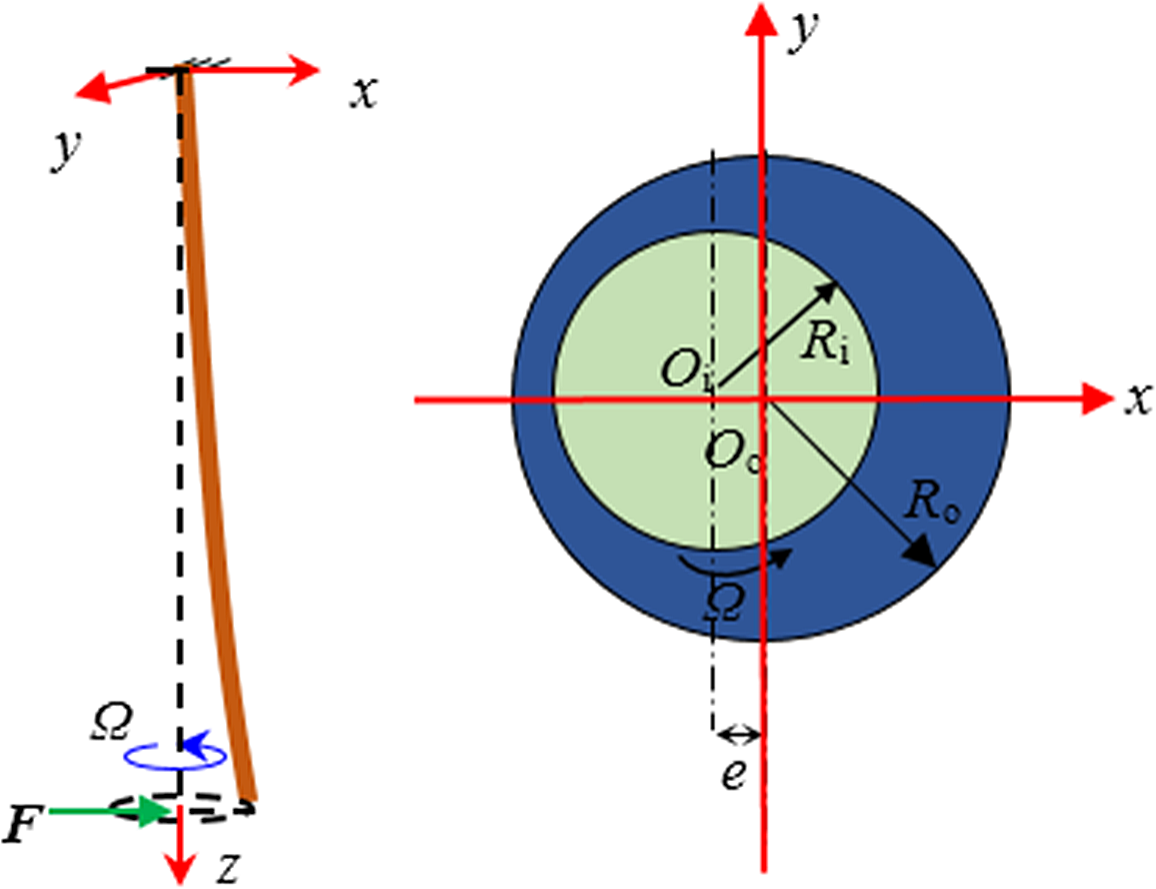
The motion of the casings in a well. When the SSFVC downhole tool is used, the casings revolve around the borehole axis.

Cementing represents an important and difficult problem in well drilling design, directly affecting the subsequent well production [1]. Many researchers have studied the flow characteristics in the annulus [2–17], including the two-phase displacement feature [18–26], and the flow in the annulus when the inner tube is in a state of motion [27–30]. Field tests have shown that vibration cementing techniques can improve the cementing quality. However, research on the reliability of the SSFVC method is insufficient [31–32]. The motion of the casings when using the SSFVC downhole tool (Fig 1) is shown in Fig 2. The main components of the SSFVC downhole tool include: (1) the bearing tray with 6 guide holes; (2) the eccentric block mounted on the outer side of the device, causing the whole device to deviate from the borehole center; (3) the mandrel (4) the blades. The tool, which is directly connected with the casing and is placed above the casing shoe, is lowered into the well with the casing string. In the process of cementing, the circulating cementing fluid, which enters the eccentric cascade through the guide holes of the bearing tray, acts on the blades on the mandrel and drives the blades to rotate at high speed. The operating frequency of the SSFVC downhole tool is 25~45 Hz. Due to the presence of the eccentric block, the bottom of the casing string is affected by an exciting force with direction changing periodically, making the casing string execute an eccentric revolution. It produces strong agitation action to the annulus fluid, makes it in the state of shear turbulent flow, and results in the formation of pulse pressure in the annular flow field. This paper presents the results of the calculation of the pulse pressure in an annular turbulent flow field, and the analysis of the effect of pulse pressure on the borehole stability.

## Mathematical model

### Governing equations

In this paper, we use the *k*-*ε* two equations turbulent model, which is the most widely used and successful model for engineering calculations of turbulent flow. The general form of the governing equations in the moving bipolar coordinates is:

$$\frac{\rho }{J}\left[\frac{\partial \left(U\phi \right)}{\partial \xi }+\frac{\partial \left(V\phi \right)}{\partial \zeta }\right]=\frac{1}{J}\frac{\partial }{\partial \xi }\left[\frac{\Gamma }{J}\left(\alpha \phi_{\xi }-\beta \phi_{\zeta }\right)\right]+\frac{1}{J}\frac{\partial }{\partial \zeta }\left[\frac{\Gamma }{J}\left(-\beta \phi_{\xi }+\gamma \phi_{\zeta }\right)\right]+S$$ (1)

$$J=x_{\xi }y_{\zeta }-x_{\zeta }y_{\xi }=-C^{2}\frac{1}{{\left(\cosh\xi -\cos\zeta \right)}^{2}}$$ (2)

$$U=uy_{\zeta }-vx_{\zeta }\;V=vx_{\xi }-uy_{\xi }$$ (3)

$$\alpha =x_{\zeta }^{2}+y_{\zeta }^{2}\;\beta =x_{\xi }x_{\zeta }-y_{\xi }y_{\zeta }\;\gamma =x_{\xi }^{2}+y_{\xi }^{2}$$ (4)

The relations between the bipolar coordinates and the rectangular coordinates are:

$$x=C\frac{\sinh\xi }{\cosh\xi -\cos\zeta }$$ (5)

$$y=C\frac{\sin\zeta }{\cosh\xi -\cos\zeta }$$ (6)

where,

$$C=\frac{{\left[{\left(R_{i}^{2}+R_{o}^{2}-e^{2}\right)}^{2}-4R_{i}^{2}R_{o}^{2}\right]}^{\frac{1}{2}}}{2e}$$ (7)

$$R_{i}=-\frac{C}{\sinh\xi_{i}}$$ (8)

$$R_{o}=-\frac{C}{\sinh\xi_{o}}$$ (9)

Thus:

$$x_{\xi }=\frac{\partial x}{\partial \xi }=C\frac{1-\cosh\xi \cos\zeta }{{\left(\cosh\xi -\cos\zeta \right)}^{2}}$$ (10)

$$x_{\zeta }=\frac{\partial x}{\partial \zeta }=C\frac{-\sinh\xi \cdot \sin\zeta }{{\left(\cosh\xi -\cos\zeta \right)}^{2}}$$ (11)

$$y_{\xi }=\frac{\partial y}{\partial \xi }=C\frac{-\sin\zeta \sinh\xi }{{\left(\cosh\xi -\cos\zeta \right)}^{2}}$$ (12)

$$y_{\zeta }=\frac{\partial y}{\partial \zeta }=C\frac{\cos\zeta \cosh\xi -1}{{\left(\cosh\xi -\cos\zeta \right)}^{2}}$$ (13)

$$C=\frac{{\left[{\left(R_{i}^{2}+R_{o}^{2}-e^{2}\right)}^{2}-4R_{i}^{2}R_{o}^{2}\right]}^{\frac{1}{2}}}{2e}$$ (14)

$$\xi_{i}=\mathrm{arcsinh}\left(-\frac{C}{R_{i}}\right)=\ln\left[-\frac{C}{R_{i}}+\sqrt{{\left(\frac{C}{R_{i}}\right)}^{2}+1}\right]$$ (15)

$$\xi_{o}=\mathrm{arcsinh}\left(-\frac{C}{R_{o}}\right)=\ln\left[-\frac{C}{R_{o}}+\sqrt{{\left(\frac{C}{R_{o}}\right)}^{2}+1}\right]$$ (16)

where, *φ* is a common variable (scalar), *ξ* (ranging from -∞ to +∞) and *ζ* (ranging from 0 to 2π) are the components for the bipolar coordinates, *U* is the velocity component in the *ξ* direction, and *V* is the velocity component in the *ζ* direction.

The governing equations of shear turbulent flow in annulus on the basis of the casing executing an eccentric revolution in the moving bipolar coordinates are shown in Table 1. There is a coupling relation between the equations. So each equation can not be solved one by one, but it must be solved simultaneously.

**Table 1:** Governing equations in moving bipolar coordinates.

	*φ*	*Γ*	*S*
Continuity equation	1	0	0
*ξ* equation	*u*	*η*_eff_ = *η*+*η*_t_	*S*_u_
*ζ* equation	*v*	*η*_eff_ = *η*+*η*_t_	*S*_v_
*w equation*	*w*	*η*_eff_ = *η*+*η*_t_	*S*_w_
*k* equation	*k*	*η*+*η*_t_ /*δ*_k_	*S*_k_
*ε* equation	*ε*	*η*+*η*_t_ /*δ*_ε_	*S*_ε_

where,

$$S_{u}^{}=-\frac{\partial p_{eff}}{\partial x}-\rho \left(L\Omega^{2}-2\Omega v-\Omega^{2}x\right)=-\frac{1}{J}p_{eff}{}_{\xi }y_{\zeta }+\frac{1}{J}p_{eff}{}_{\zeta }y_{\xi }-\rho \left(L\Omega^{2}-2\Omega v-\Omega^{2}C\frac{\sinh\xi }{\cosh\xi -\cos\zeta }\right)$$ (17)

$$S_{v}^{}=-\frac{\partial p_{eff}}{\partial y}-\rho \left(2\Omega u-\Omega^{2}y\right)=\frac{1}{J}p_{eff}{}_{\xi }x_{\zeta }-\frac{1}{J}p_{eff}{}_{\zeta }x_{\xi }-\rho \left(2\Omega u-\Omega^{2}C\frac{\sin\zeta }{\cosh\xi -\cos\zeta }\right)$$ (18)

$$S_{w}^{}=-\rho g-\frac{\partial p}{\partial z}$$ (19)

$$S_{k}^{}=\eta_{t}\{2\left[{\left(\frac{\partial u}{\partial \xi }\frac{y_{\zeta }}{J}-\frac{\partial u}{\partial \zeta }\frac{y_{\xi }}{J}\right)}^{2}+{\left(-\frac{\partial v}{\partial \xi }\frac{x_{\zeta }}{J}+\frac{\partial v}{\partial \zeta }\frac{x_{\xi }}{J}\right)}^{2}\right]+{\left(\frac{\partial u}{\partial \xi }\frac{y_{\zeta }}{J}-\frac{\partial u}{\partial \zeta }\frac{y_{\xi }}{J}-\frac{\partial v}{\partial \xi }\frac{x_{\zeta }}{J}+\frac{\partial v}{\partial \zeta }\frac{x_{\xi }}{J}\right)}^{2}+{\left(\frac{\partial w}{\partial \xi }\frac{y_{\zeta }}{J}-\frac{\partial w}{\partial \zeta }\frac{y_{\xi }}{J}\right)}^{2}+{\left(-\frac{\partial w}{\partial \xi }\frac{x_{\zeta }}{J}+\frac{\partial w}{\partial \zeta }\frac{x_{\xi }}{J}\right)}^{2}\}-\rho \varepsilon$$ (20)

$$\begin{array}{l} S_{\varepsilon }^{}=\frac{\varepsilon }{k}\{C_{1\varepsilon }\eta_{t}\{2\left[{\left(\frac{\partial u}{\partial \xi }\frac{y_{\zeta }}{J}-\frac{\partial u}{\partial \zeta }\frac{y_{\xi }}{J}\right)}^{2}+{\left(-\frac{\partial v}{\partial \xi }\frac{x_{\zeta }}{J}+\frac{\partial v}{\partial \zeta }\frac{x_{\xi }}{J}\right)}^{2}\right]+{\left(-\frac{\partial u}{\partial \xi }\frac{x_{\zeta }}{J}+\frac{\partial u}{\partial \zeta }\frac{x_{\xi }}{J}+\frac{\partial v}{\partial \xi }\frac{y_{\zeta }}{J}-\frac{\partial v}{\partial \zeta }\frac{y_{\xi }}{J}\right)}^{2}\\ \;+{\left(\frac{\partial w}{\partial \xi }\frac{y_{\zeta }}{J}-\frac{\partial w}{\partial \zeta }\frac{y_{\xi }}{J}\right)}^{2}+{\left(-\frac{\partial w}{\partial \xi }\frac{x_{\zeta }}{J}+\frac{\partial w}{\partial \zeta }\frac{x_{\xi }}{J}\right)}^{2}\}-C_{2\varepsilon }\rho \varepsilon \} \end{array}$$ (21)

$$\eta =\eta \left(I_{2}\right)=K{\left(I_{2}\right)}^{n-1}$$ (22)

$$\begin{array}{l} I_{2}={\left\{\frac{1}{2}trA_{1}^{'}{}^{2}\right\}}^{\frac{1}{2}}=\{2{\left(\frac{\partial u}{\partial \xi }\frac{y_{\zeta }}{J}-\frac{\partial u}{\partial \zeta }\frac{y_{\xi }}{J}\right)}^{2}+2{\left(-\frac{\partial v}{\partial \xi }\frac{x_{\zeta }}{J}+\frac{\partial v}{\partial \zeta }\frac{x_{\xi }}{J}\right)}^{2}\\ \;+{\left(\frac{\partial v}{\partial \xi }\frac{y_{\zeta }}{J}-\frac{\partial v}{\partial \zeta }\frac{y_{\xi }}{J}-\frac{\partial u}{\partial \xi }\frac{x_{\zeta }}{J}+\frac{\partial u}{\partial \zeta }\frac{x_{\xi }}{J}\right)}^{2}{+{\left(\frac{\partial w}{\partial \xi }\frac{y_{\zeta }}{J}-\frac{\partial w}{\partial \zeta }\frac{y_{\xi }}{J}\right)}^{2}+{\left(-\frac{\partial w}{\partial \xi }\frac{x_{\zeta }}{J}+\frac{\partial w}{\partial \zeta }\frac{x_{\xi }}{J}\right)}^{2}\}}^{\frac{1}{2}} \end{array}$$ (23)

$$p_{eff}=p-\frac{2}{3}\rho k$$ (24)

$$\eta_{t}=C_{\mu }\frac{\rho k^{2}}{\varepsilon }$$ (25)

$$C_{\mu }=0.09\;C_{1\varepsilon }^{}=1.44\;C_{2\varepsilon }^{}=1.92$$ (26)

$$\delta_{k}=1.0\;\delta_{\varepsilon }=1.3$$ (27)

### Boundary conditions

The boundary conditions in the moving bipolar coordinates during SSFVC are shown in Table 2.

**Table 2:** Boundary conditions in moving bipolar coordinates.

	Boundary conditions
Boundary conditions for velocity	${\left(-vx_{\xi }+uy_{\xi }\right)|}_{\xi =\xi_{i}}=0\;{\left(-vx_{\xi }+uy_{\xi }\right)|}_{\xi =\xi_{o}}=\sqrt{J}\Omega R_{o}$ ${\left(-vx_{\zeta }+uy_{\zeta }\right)|}_{\xi =\xi_{i}}=0\;{\left(-vx_{\zeta }+uy_{\zeta }\right)|}_{\xi =\xi_{o}}=0$ ${w|}_{\xi =\xi_{i}}=0\;{w|}_{\xi =\xi_{o}}=0$
Boundary conditions for *k* and *ε*	$\frac{\partial k}{\partial n}=0\;\varepsilon_{P}=\frac{C_{\mu }^{3/4}k_{P}^{3/2}}{\kappa y_{P}}$
Inlet boundary condition for velocity	$w=\frac{Q}{\pi \left(R_{o}^{2}-R_{i}^{2}\right)}$
Inlet boundary conditions for *k* and *ε*	$k=\frac{1}{2}\alpha_{k}w^{2}\;\alpha_{k}=0.5\%\sim 1.5\%\;\varepsilon =C_{\mu }k^{3/2}/l$
Outlet boundary conditions for *k* and *ε*	$\frac{\partial k}{\partial n}=0\;\frac{\partial \varepsilon }{\partial n}=0$

### Influence of vibration on surrounding rock stress

Borehole instability is one of the common borehole problems. The surrounding rock stress under the effect of pulse pressure during SSFVC is different from that during the conventional cementing. It is more prone to cause borehole problems, such as lost circulation and even formation fracturing. Therefore, determination of surrounding rock stress is of great importance to avoid borehole problems. The pulse pressure results in the formation of a stress wave, which affects the surrounding rock stress. The attenuation law of vibration stress in the rock *p*_sw_ with regard to distance is:

$$p_{sw}=P_{sw}{\left(\frac{r_{}^{2}}{R_{o}^{2}}\right)}^{-\alpha }$$ (28)

$$\alpha =-4.11\times 10^{-7}\times \rho_{r}C_{p}+2.92$$ (29)

$$C_{p}=1.68-0.0002*H$$ (30)

where, *P*_sw_ is the vibration stress at the borehole wall, *r* is the distance from the center of the borehole, *α* is the pressure attenuation index, *ρ*_r_ is the rock density, *C*_p_ is the rock compaction correction coefficient, and *H* is the formation depth.

Since a large wave impedance contrast exists between the fluid and the rock, the stress wave exhibits a refraction phenomenon at the interface. The formula for wave impedance is:

I=ρV (31)

According to the commonly used Gardner empirical formula:

$$\rho =aV^{n}$$ (32)

where, *V* is the propagation velocity of the stress wave, *ρ* is the medium density, *a* = 0.31, *n* = 0.25. Then,

$$\rho ={\left(aI^{n}\right)}^{\frac{1}{n+1}}$$ (33)

*k* is the wave impedance ratio of the rock and the fluid, so:

$$k=\frac{I_{r}}{I_{l}}=\frac{{\left(\frac{\rho_{r}^{n+1}}{a}\right)}^{1/n}}{{\left(\frac{\rho_{l}^{n+1}}{a}\right)}^{1/n}}={\left(\frac{\rho_{r}^{}}{\rho_{l}^{}}\right)}^{1+\frac{1}{n}}$$ (34)

Thus,

$$P_{sw}=kP$$ (35)

where, *P* is the pulse pressure in the annular flow field.

According to the theory of porous elastic medium mechanics and considering the effect of pulse pressure, the effective stresses of the rock at the distance *r* from the center of the borehole are:

$$\left\{ \begin{array}{l} \sigma_{r}=\frac{R_{o}^{2}}{r^{2}}P_{w}+\frac{\left(\sigma_{H}+\sigma_{h}\right)}{2}(1-\frac{R_{o}^{2}}{r^{2}})+\frac{\left(\sigma_{H}-\sigma_{h}\right)}{2}(1+\frac{3R_{o}^{4}}{r^{4}}-\frac{4R_{o}^{2}}{r^{2}})\cos2\theta -\gamma P\left(r\right)+kP{\left(\frac{r_{}^{2}}{R_{o}^{2}}\right)}^{-\alpha }\\ \sigma_{\theta }=-\frac{R_{o}^{2}}{r^{2}}P_{w}+\frac{\left(\sigma_{H}+\sigma_{h}\right)}{2}(1+\frac{R_{o}^{2}}{r^{2}})-\frac{\left(\sigma_{H}-\sigma_{h}\right)}{2}(1+\frac{3R_{o}^{4}}{r^{4}})\cos2\theta -\gamma P\left(r\right)-kP{\left(\frac{r_{}^{2}}{R_{o}^{2}}\right)}^{-\alpha }\\ \sigma_{z}=\sigma_{v}-\nu \left[2\left(\sigma_{H}-\sigma_{h}\right)\frac{R_{o}^{2}}{r^{2}}\cos2\theta \right]-\gamma P\left(r\right)\\ \tau_{r\theta }=\frac{\left(\sigma_{H}-\sigma_{h}\right)}{2}(1-\frac{3R_{o}^{4}}{r^{4}}+\frac{2R_{o}^{2}}{r^{2}})\sin2\theta \end{array}\right.$$ (36)

where, *σ*_r_, *σ*_θ_, *σ*_z,_ and *τ*_rθ_ are, respectively, the effective radial stress, effective tangential stress, effective vertical pressure, and shear stress, *σ*_H_ is the maximum horizontal stress, *σ*_h_ is the minimum horizontal stress, *P*_w_ is the bottom hole pressure, *γ* is the effective stress coefficient, and *P*(*r*) is the pore pressure at the distance *r* from the center of the borehole.

At the borehole wall, *r* = *R*_o_, *P*(*r*) = *P*_p_, the effective stresses are:

$$\left\{ \begin{array}{l} \sigma_{r}=P_{w}-\gamma P_{p}+kP\\ \sigma_{\theta }=-P_{w}+\left(\sigma_{H}+\sigma_{h}\right)-2\left(\sigma_{H}-\sigma_{h}\right)\cos2\theta -\gamma P_{p}-kP\\ \sigma_{z}=\sigma_{v}-2\nu \left(\sigma_{H}-\sigma_{h}\right)\cos2\theta -\gamma P_{p}\\ \tau_{r\theta }=0 \end{array}\right.$$ (37)

where, *P*_p_ is the formation pressure.

So far, the calculation model of the effective stresses of the rock around a borehole considering the effect of pulse pressure has been established. And according to the rock strength, the critical condition for ensuring the borehole stability can be determined.

According to the Mohr-Coulomb criterion:

$$\sigma_{1}=m\sigma_{3}+\sigma_{c}$$ (38)

$$m=\frac{1+\sin\varphi }{1-\sin\varphi }$$ (39)

$$\sigma_{c}=\frac{2C\cdot \cos\varphi }{1-\sin\varphi }$$ (40)

The formula for collapse pressure:

$$P_{c}=\frac{\left(3\sigma_{H}-\sigma_{h}\right)+\gamma \left(m-1\right)P_{p}-\left(1+m\right)kP-\sigma_{c}}{m+1}$$ (41)

According to the Minimum Principal Stress Destroy criterion:

$$\sigma_{\theta }=-S_{t}$$ (42)

The formula for fracture pressure:

$$P_{f}=\left(3\sigma_{h}-\sigma_{H}\right)-\gamma P_{p}-kP+S_{t}$$ (43)

In the process of vibration cementing, complicated downhole accidents, such as lost circulation and even formation fracturing, are likely to occur under the effect of pulse pressure. Based on the calculation formula (43) of formation fracture pressure, the critical pulse pressure *P*_cv_ without formation fracturing is:

$$P_{cv}=\frac{\left(3\sigma_{h}-\sigma_{H}\right)-\gamma P_{p}-P_{f}+S_{t}}{k}$$ (44)

By formula (44), the critical pulse pressure *P*_cv_ without formation fracturing can be obtained on the basis of parameters such as formation stress, rock strength and fracture pressure.

## Results and discussion

**Fig 3:**
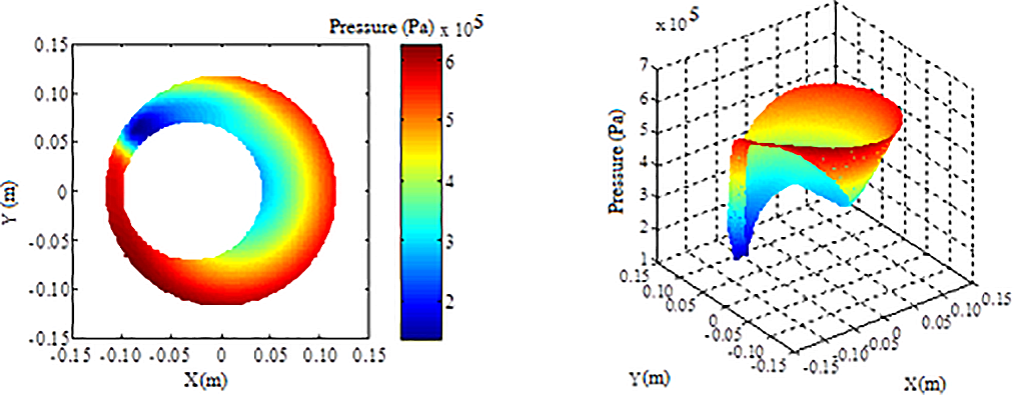
The pressure distribution at *δ* = 0.6 with *Ω* = 2000r/min.

**Fig 4:**
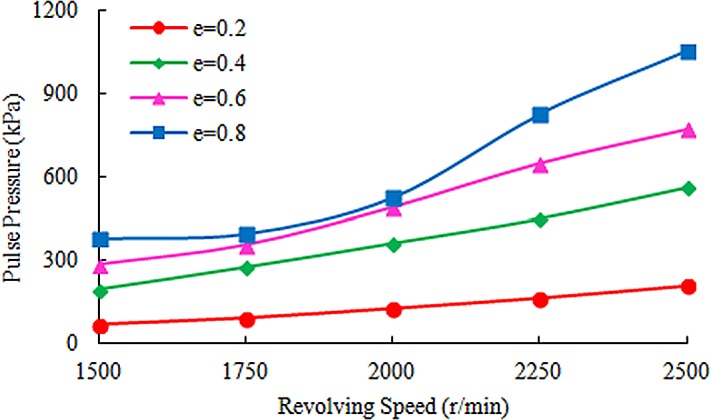
Relationship between pulse pressure and revolving speed with different eccentricity.

On the basis of governing equations and boundary conditions of shear turbulent flow in annulus, the pressure distribution was obtained with the finite volume method, as shown in Fig 3, taking *δ* = 0.6 and *Ω* = 2000r/min as an example. As can be seen from Fig 3, when fluid flowed from the wide clearance to the narrow clearance, due to the existence of low pressure area near the narrow clearance, high-pressure fluid near the wide clearance tended to flow to the low pressure area and displaced the fluid near the narrow clearance, which helped to reduce the detention. When the fluid flowed from the narrow clearance to the wide clearance, the pressure decreased gradually from narrow clearance to wide clearance, which was beneficial to the flow of the fluid in the narrow clearance. It also helped to reduce the detention. And the relationship between pulse pressure and revolving speed with different eccentricity was obtained by calculating the pressure distribution under different conditions, as shown in Fig 4. The pulse pressure could cause the annulus fluid to vibrate, which was beneficial to improve the liquidity and reduce the retention. It could be seen that greater revolving speed could guarantee better cementing quality from the bottom hole.

The influence of the vibration on the gel strength of drilling fluid and cement slurry was studied by laboratory tests with the six-speed rotary viscosimeter. The experimental results were shown in Tables 3 and 4. It could be seen that the gel strength of the fluid became small because the vibration would help to break the bonding between the particles in the fluid. The influence of the vibration on the drilling fluid was conducive to reduce its viscosity and the retention. The gel strength decrease of the cement slurry would delay the decrease of hydrostatic pressure in annulus and prevent the formation fluid from channeling, thereby improving the cementing quality.

**Table 3:** Measurement for gel strength of drilling fluid.

Cementing conditions	The reading of six-speed rotary viscosimeter *φ*3		Gel strength (Pa)		Drop rate of gel strength (%)	
	Stand 1min	Stand 10min	Initial gel (Pa)	Final gel (Pa)	Drop rate of initial gel (%)	Drop rate of final gel (%)
Conventional cementing	12	26	6.612	14.326	/	/
1500r/min	11	23	6.061	12.673	8	12
1750r/min	10	21	5.51	11.571	17	19
2000r/min	9	19	4.959	10.469	25	27
2250r/min	8	17	4.408	9.367	33	35
2500r/min	7	16	3.857	8.816	42	38

**Table 4:** Measurement for gel strength of cement slurry.

Cementing conditions	The reading of six-speed rotary viscosimeter *φ*3		Gel strength (Pa)		Drop rate of gel strength (%)	
	Stand 1min	Stand 10min	Initial gel (Pa)	Final gel (Pa)	Drop rate of initial gel (%)	Drop rate of final gel (%)
Conventional cementing	17	51	9.367	28.101	/	/
1500r/min	16	48	8.816	26.448	6	6
1750r/min	15	46	8.265	25.346	12	10
2000r/min	14	45	7.714	24.795	18	12
2250r/min	13	43	7.163	23.693	24	16
2500r/min	12	42	6.612	23.142	29	18

According to the revolving speed calculation model of the downhole eccentric cascade[33], the revolving speed depended on the displacement in the case of certain structural parameters of the downhole eccentric cascade. That was to say, the revolving speed would be optimizd by the critical pulse pressure to ensure the cementing safety, which could guide the cementing operation.

**Fig 5:**
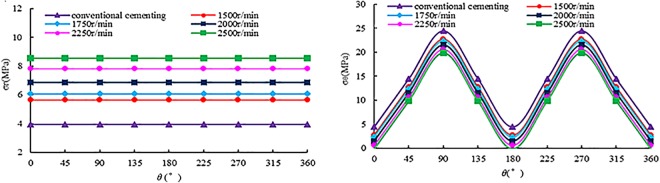
The distribution of σ_r_ and σ_θ_ at different revolving speeds for SSFVC and conventional cementing.

The eccentricity was taken as 0.6, because the wall shear stress was great enough to improve displacement efficiency at *e* = 0.6 [34] and it was advantageous to avoid the occurrence of collision. Based on the basic parameters in Table 5, the effective stresses at the borehole wall were calculated, with the details shown in Fig 5. As can be seen from Fig 5, the effective radial stress at the borehole wall during SSFVC was greater than that during conventional cementing; the faster the revolving speeds, the greater was the effective radial stress. The effective tangential stress at the borehole wall during SSFVC was smaller than that during conventional cementing; the faster the revolving speeds, the smaller was the effective tangential stress.

**Table 5:** Basic parameters.

Basic Parameters	Value
Borehole radius (m)	0.118
Casing radius (m)	0.06985
Bottom hole pressure (MPa)	26.25
Formation pressure (MPa)	22.3
Fluid density (kg·m^-3^)	1750
Internal friction of rock (°)	10
Rock cohesion (MPa)	18
Well depth (m)	1500
Rock density (kg·m^-3^)	2500
Effective stress coefficient	1.0
Poisson ratio	0.2
Tensile strength (MPa)	4.59
The maximum horizontal stress (MPa)	34
The minimum horizontal stress (MPa)	29
Collapse pressure(MPa)	17.8
Fracture Pressure(MPa)	32.5

According to the basic parameters (as shown in Table 5), the critical pulse pressure without formation fracturing calculated by formula (44) was 468.6 kPa. As shown in Fig 4, the pulse pressure was 467.3kPa when the eccentricity was equal to 0.6 and the revolving speed was equal to 2000r/min. So in order to ensure borehole stability during SSFVC, the revolving speed should not exceed 2000r/min. Based on the revolving speed calculation model of the downhole eccentric cascade, the revolving speed was 1950r/min when the displacement was 1.8m^3^/min. In the case, not only the cementing safety would be ensured, but also the cementing quality could be improved to the most extent.

## Conclusion

1. Governing equations and boundary conditions of the annular turbulent flow field in moving bipolar coordinates were derived.
2. This study established a calculation model of the effective stresses of the rock around a borehole considering the effect of pulse pressure.
3. According to the Mohr-Coulomb criterion and the Minimum Principal Stress Destroy criterion, this paper deduced the formulas for collapse pressure and fracture pressure. The critical condition for ensuring borehole stability was obtained.
4. The pulse pressure is conducive to improving the cementing quality. However, the pulse pressure changes the surrounding rock stress and may threaten the borehole stability. Lost circulation is more likely to occur for the SSFVC method. In order to ensure safe procedures during SSFVC, reasonably optimized revolving speed has to be ensured, which can guiding the cementing operation.

## Supporting information

S1 File The derivation of the governing equations of shear turbulent flow in annulus on the basis of the casing executing an eccentric revolution. (DOC)

## Nomenclature

R_i_: the inner diameter of the annulus
*R*_o_: the outer diameter of the annulus
e: eccentricity
Ω: revolving speed
U: the velocity components in the *ξ* direction in the computational space
V: the velocity components in the *ζ* direction in the computational space
φ: a common variable (scalar)
η: fluid viscosity
η_t_: turbulent viscosity
P: the pulse pressure in the borehole
r: the distance from the center of the borehole
α: the pressure attenuation index
*ρ*_r_: rock density
*C*_p_: the rock compaction correction coefficient
H: formation depth
*σ*_r_: effective radial stress
*σ*_θ_: effective tangential stress
*σ*_z_: effective vertical pressure
*τ*_rθ_: shear stress
*σ*_H_: the maximum horizontal stress
*σ*_h_: the minimum horizontal stress
*P*_w_: bottom hole pressure
γ: the effective stress coefficient
*P*(*r*): the pore pressure at the distance *r* from the center of the borehole
*P*_p_: formation pressure
*P*_c_: collapse pressure
*P*_f_: fracture pressure
*p*_sw_: vibration stress in the rock
*P*_sw_: vibration stress at the borehole wall
*P*_cv_: the critical pulse pressure
