# Supplementary material for: Effect of pulse pressure on borehole stability during shear swirling flow vibration cementing
Source: PLoS One. 2017 Nov 16;12(11):e0187279. doi: 10.1371/journal.pone.0187279 (PMC5690648; doi:10.1371/journal.pone.0187279)
Supplement: S1 File — (DOC) [file pone.0187279.s001.doc]

**The derivation of the control equations of shear turbulent flow in annulus on the basis of the casing executing an eccentric revolution**

(1)The continuity equation of incompressible fluid in rectangular coordinates is:

$\frac{\partial u}{\partial x}+\frac{\partial v}{\partial y}+\frac{\partial w}{\partial z}=0$ (1)

where, *u*, *v*, *w* respectively are the components of the velocity ***v*** in *x*, *y*, *z* direction.

In the case of axisymmetric flow, the continuity equation is

$\frac{\partial u}{\partial x}+\frac{\partial v}{\partial y}=0$ (2)

The relationship between the velocity in the inertial rectangular coordinates and the moving rectangular coordinates is:

$\mathbf{v}=-L\Omega\mathbf{j}'+\mathbf{v}'+\Omega\times\mathbf{r}'$ (3)

The relationship between the acceleration in the inertial rectangular coordinates and the moving rectangular coordinates is:

$\mathbf{a}=\mathbf{a}_{o'}+\mathbf{a}'+2\Omega\times\mathbf{v}'+\Omega\times\left(\Omega\times r'\right)$ (4)

In the inertial rectangular coordinates, the tensor expression of N-S equation is

$\rho\frac{d\mathbf{v}}{dt}=\rho\mathbf{f}-grad\mathbf{p}+div\mathbf{T}$ (5)

Substituting formula (4) in formula (5), the N-S equation in the moving rectangular coordinates is:

$\rho\mathbf{a}'=\rho\left[\mathbf{f}-\mathbf{a}_{o'}-2\Omega\times\mathbf{v}'-\Omega\times\left(\Omega\times r'\right) \right]-grad\mathbf{p}+div'\mathbf{T}$ (6)

where,

$div'=\frac{\partial}{\partial x'}\mathbf{i}'+\frac{\partial}{\partial y'}\mathbf{j}'+\frac{\partial}{\partial z'}\mathbf{k}'$ (7)

$\mathbf{a}'=\frac{\partial'\mathbf{v}'}{\partial t}+\left(\mathbf{v}'\cdot\mathrm{div}\right)\mathbf{v}'$ (8)

The power law constitutive model is:

$T=\eta A_{1}$ (9)

Component equations are:

$T_{xx}=2\eta\frac{\partial u}{\partial x}$ (10)

$T_{xy}=T_{yx}=\eta(\frac{\partial v}{\partial x}+\frac{\partial u}{\partial y})$ (11)

$T_{xz}=T_{zx}=\eta\frac{\partial w}{\partial x}$ (12)

$T_{yy}=2\eta\frac{\partial v}{\partial y}$ (13)

$T_{yz}=T_{zy}=\eta\frac{\partial w}{\partial y}$ (14)

$T_{zz}=0$ (15)

The power law constitutive model is used to substitute the components of the N-S equation in the moving rectangular coordinates without considering *f*(*x*) and *f*(*y*). Thus,

$-\frac{\partial p}{\partial x}+2\frac{\partial}{\partial x}\left(\eta\frac{\partial u}{\partial x}\right)+\frac{\partial}{\partial y}\left[\eta(\frac{\partial v}{\partial x}+\frac{\partial u}{\partial y})\right]=\rho\left(u\frac{\partial u}{\partial x}+v\frac{\partial u}{\partial y}+L\Omega^{2}-2\Omega v-\Omega^{2}x\right)$ (16)

$-\frac{\partial p}{\partial y}+\frac{\partial}{\partial x}\left[\eta(\frac{\partial v}{\partial x}+\frac{\partial u}{\partial y})\right]+2\frac{\partial}{\partial y}\left(\eta\frac{\partial v}{\partial y}\right)=\rho\left(u\frac{\partial v}{\partial x}+v\frac{\partial v}{\partial y}+2\Omega u-\Omega^{2}y\right)$ (17)

$-\rho g-\frac{\partial p}{\partial z}+\frac{\partial}{\partial x}\left(\eta\frac{\partial w}{\partial x}\right)+\frac{\partial}{\partial y}\left(\eta\frac{\partial w}{\partial y}\right)=\rho\left(u\frac{\partial w}{\partial x}+v\frac{\partial w}{\partial y}\right)$ (18)

(2)According to the time average method and the average operation law, the continuity equation of shear turbulent flow of incompressible flow is expressed as:

$\frac{\overline{\partial u}}{\partial x}+\frac{\overline{\partial v}}{\partial y}=\frac{\overline{\partial\left(\overline{u}+u'\right)}}{\partial x}+\frac{\overline{\partial\left(\overline{v}+v'\right)}}{\partial y} = \frac{\overline{\partial\overline{u}}}{\partial x}+ \frac{\overline{\partial u'}}{\partial x}+ \frac{\overline{\partial\overline{v}}}{\partial y}+ \frac{\overline{\partial v'}}{\partial y} = \frac{\partial\overline{u}}{\partial x}+ \frac{\partial\overline{u'}}{\partial x}+ \frac{\partial\overline{v}}{\partial y}+ \frac{\partial\overline{v'}}{\partial y} =0$ (19)

where:

$\overline{u'}=0$ (20)

$\overline{v'}=0$ (21)

Then

$\frac{\partial\overline{u}}{\partial x}+ \frac{\partial\overline{v}}{\partial y}=0$ (22)

Based on the time average method, then

$\frac{\partial\left(\overline{u}+u'\right)}{\partial x}=\frac{\partial\overline{u}}{\partial x}+\frac{\partial u'}{\partial x}$ (23)

$\frac{\partial\left(\overline{v}+v'\right)}{\partial y}=\frac{\partial\overline{v}}{\partial y}+\frac{\partial v'}{\partial y}$ (24)

Thus:

$\frac{\partial u'}{\partial x}+ \frac{\partial v'}{\partial y}=0$ (25)

According to the time average method and the average operation law, the equation (16) is expressed as:

$\begin{matrix} -\frac{\partial\left(\overline{p}+p'\right)}{\partial x}+2\frac{\partial}{\partial x}\left[\eta\frac{\partial\left(\overline{u}+u'\right)}{\partial x}\right]+\frac{\partial}{\partial y}\left[\eta\frac{\partial\left(\overline{v}+v'\right)}{\partial x}+\eta\frac{\partial\left(\overline{u}+u'\right)}{\partial y}\right] \\ =\rho\left[\left(\overline{u}+u'\right)\frac{\partial\left(\overline{u}+u'\right)}{\partial x}+\left(\overline{v}+v'\right)\frac{\partial\left(\overline{u}+u'\right)}{\partial y}+L\Omega^{2}-2\Omega v-\Omega^{2}x\right] \end{matrix}$ (26)

Eliminate items which are equal to zero.Then

$\begin{matrix} -\frac{\partial\overline{p}}{\partial x}+2\frac{\partial}{\partial x}\left(\eta\frac{\partial\overline{u}}{\partial x}\right)+\frac{\partial}{\partial y}\left(\eta\frac{\partial\overline{v}}{\partial x}+\eta\frac{\partial\overline{u}}{\partial y}\right) \\ =\rho\left(\overline{u}\frac{\partial\overline{u}}{\partial x}+\overline{u'\frac{\partial u'}{\partial x}}+\overline{v}\frac{\partial\overline{u}}{\partial y}+\overline{v'\frac{\partial u'}{\partial y}}+L\Omega^{2}-2\Omega\overline{v}-\Omega^{2}x\right) \end{matrix}$ (27)

where,

$\overline{u'\frac{\partial u'}{\partial x}}=\overline{\frac{\partial}{\partial x}\left(u'u'\right)}-\overline{u'\frac{\partial u'}{\partial x}}$ (28)

$\overline{v'\frac{\partial u'}{\partial y}}=\overline{\frac{\partial}{\partial y}\left(u'v'\right)}-\overline{u'\frac{\partial v'}{\partial y}}$ (29)

Then

$\begin{matrix} -\frac{\partial\overline{p}}{\partial x}+2\frac{\partial}{\partial x}\left(\eta\frac{\partial\overline{u}}{\partial x}\right)+\frac{\partial}{\partial y}\left(\eta\frac{\partial\overline{v}}{\partial x}+\eta\frac{\partial\overline{u}}{\partial y}\right) \\ =\rho\left[\overline{u}\frac{\partial\overline{u}}{\partial x}+\overline{\frac{\partial}{\partial x}\left(u'u'\right)}-\overline{u'\frac{\partial u'}{\partial x}}+\overline{v}\frac{\partial\overline{u}}{\partial y}+\overline{\frac{\partial}{\partial y}\left(u'v'\right)}-\overline{u'\frac{\partial v'}{\partial y}}+L\Omega^{2}-2\Omega\overline{v}-\Omega^{2}x\right] \\ =\rho\left[\overline{u}\frac{\partial\overline{u}}{\partial x}+\overline{v}\frac{\partial\overline{u}}{\partial y}+\overline{\frac{\partial}{\partial x}\left(u'u'\right)}+\overline{\frac{\partial}{\partial y}\left(u'v'\right)}-\left(\overline{u'\frac{\partial u'}{\partial x}+u'\frac{\partial v'}{\partial y}}\right)+L\Omega^{2}-2\Omega\overline{v}-\Omega^{2}x\right] \end{matrix}$ (30)

For convenience, in addition to the mean value of the pulsation value, the overlined symbol of the mean value is removed below. Then

$\frac{\partial u}{\partial x}+ \frac{\partial v}{\partial y}=0$ (31)

$\begin{matrix} -\frac{\partial p}{\partial x}+\frac{\partial}{\partial x}\left[2\eta\frac{\partial u}{\partial x}-\rho\left(\overline{u'u'}\right)\right]+\frac{\partial}{\partial y}\left[\left(\eta\frac{\partial v}{\partial x}\right)+\left(\eta\frac{\partial u}{\partial y}\right)-\rho\left(\overline{u'v'}\right)\right] \\ =\rho\left(u\frac{\partial u}{\partial x}+v\frac{\partial u}{\partial y}+L\Omega^{2}-2\Omega v-\Omega^{2}x\right) \end{matrix}$ (32)

Similarly,

$\begin{matrix} -\frac{\partial p}{\partial y}+\frac{\partial}{\partial x}\left[\left(\eta\frac{\partial v}{\partial x}\right)+\left(\eta\frac{\partial u}{\partial y}\right)-\rho\left(\overline{u'v'}\right)\right]+\frac{\partial}{\partial y}\left[2\left(\eta\frac{\partial v}{\partial y}\right)-\rho\left(\overline{v'v'}\right)\right] \\ =\rho\left(u\frac{\partial v}{\partial x}+v\frac{\partial v}{\partial y}+2\Omega u-\Omega^{2}y\right) \end{matrix}$ (33)

$-\rho g-\frac{\partial p}{\partial z}+\frac{\partial}{\partial x}\left(\eta\frac{\partial w}{\partial x}\right)+\frac{\partial}{\partial y}\left(\eta\frac{\partial w}{\partial y}\right)-\rho\left(\overline{\frac{\partial}{\partial x}\left(u'w'\right)}+\overline{\frac{\partial}{\partial y}\left(v'w'\right)}\right) \text{=}\rho\left(u\frac{\partial w}{\partial x}+v\frac{\partial w}{\partial y}\right)$ (34)

According to the Boussinesq momentum hypothesis:

$-\rho\overline{u_{i}^{'}u_{j}^{'}}=\eta_{t}\left(\frac{\partial u_{i}}{\partial x_{j}}+\frac{\partial u_{j}}{\partial x_{i}}\right)-\frac{2}{3}\rho k\delta_{ij}$ (35)

where, when *i*=*j*，*δ*_ij_=1; when *i*≠*j*，*δ*_ij_=0.

Then

$-\rho\left(\overline{u'u'}\right)=2\eta_{t}\frac{\partial u}{\partial x}-\frac{2}{3}\rho k$ (36)

$-\rho\left(\overline{u'v'}\right)=\eta_{t}\left(\frac{\partial u}{\partial y}+\frac{\partial v}{\partial x}\right)$ (37)

$-\rho\left(\overline{v'v'}\right)=2\eta_{t}\frac{\partial v}{\partial y}-\frac{2}{3}\rho k$ (38)

$-\rho\left(\overline{u'w'}\right)=\eta_{t}\left(\frac{\partial u}{\partial z}+\frac{\partial w}{\partial x}\right)\text{=}\eta_{t}\frac{\partial w}{\partial x}$ (39)

$-\rho\left(\overline{v'w'}\right)=\eta_{t}\left(\frac{\partial v}{\partial z}+\frac{\partial w}{\partial y}\right)\text{=}\eta_{t}\frac{\partial w}{\partial y}$ (40)

Substituting formula (36)~(40) in formula (32)~(34), then

$\begin{matrix} -\frac{\partial}{\partial x}\left(p-\frac{2}{3}\rho k\right)+\frac{\partial}{\partial x}\left[2\left(\eta+\eta_{t}\right)\frac{\partial u}{\partial x}\right]+\frac{\partial}{\partial y}\left[\left(\eta+\eta_{t}\right)\left(\frac{\partial u}{\partial y}+\frac{\partial v}{\partial x}\right)\right] \\ =\rho\left(u\frac{\partial u}{\partial x}+v\frac{\partial u}{\partial y}+L\Omega^{2}-2\Omega v-\Omega^{2}x\right) \end{matrix}$ (41)

$\begin{matrix} -\frac{\partial}{\partial y}\left(p-\frac{2}{3}\rho k\right)+\frac{\partial}{\partial x}\left[\left(\eta+\eta_{t}\right)\left(\frac{\partial u}{\partial y}+\frac{\partial v}{\partial x}\right)\right]+\frac{\partial}{\partial y}\left[2\left(\eta+\eta_{t}\right)\frac{\partial v}{\partial y}\right] \\ =\rho\left(u\frac{\partial v}{\partial x}+v\frac{\partial v}{\partial y}+2\Omega u-\Omega^{2}y\right) \end{matrix}$ (42)

$-\rho g-\frac{\partial p}{\partial z}+\frac{\partial}{\partial x}\left[\left(\eta+\eta_{t}\right)\frac{\partial w}{\partial x}\right]+\frac{\partial}{\partial y}\left[\left(\eta+\eta_{t}\right)\frac{\partial w}{\partial y}\right] \text{=}\rho\left(u\frac{\partial w}{\partial x}+v\frac{\partial w}{\partial y}\right)$ (43)

Because:

$\frac{\partial u}{\partial x}+\frac{\partial v}{\partial y}\text{=}0$ (44)

Then

$\frac{\partial}{\partial x}\left[\left(\eta+\eta_{t}\right)\frac{\partial u}{\partial x}\right]+\frac{\partial}{\partial y}\left[\left(\eta+\eta_{t}\right)\frac{\partial v}{\partial x}\right]\text{=}\frac{\partial}{\partial x}\left[\left(\eta+\eta_{t}\right)\frac{\partial u}{\partial x}\right]+\frac{\partial}{\partial x}\left[\left(\eta+\eta_{t}\right)\frac{\partial v}{\partial y}\right]\text{=}0$ (45)

$\frac{\partial}{\partial x}\left[\left(\eta+\eta_{t}\right)\frac{\partial u}{\partial y}\right]+\frac{\partial}{\partial y}\left[\left(\eta+\eta_{t}\right)\frac{\partial v}{\partial y}\right]\text{=}\frac{\partial}{\partial y}\left[\left(\eta+\eta_{t}\right)\frac{\partial u}{\partial x}\right]+\frac{\partial}{\partial y}\left[\left(\eta+\eta_{t}\right)\frac{\partial v}{\partial y}\right]\text{=}0$ (46)

Thus, Reynolds equations in *x* and *y* direction are reduced to:

$\begin{matrix} -\frac{\partial}{\partial x}\left(p-\frac{2}{3}\rho k\right)+\frac{\partial}{\partial x}\left[\left(\eta+\eta_{t}\right)\frac{\partial u}{\partial x}\right]+\frac{\partial}{\partial y}\left[\left(\eta+\eta_{t}\right)\frac{\partial u}{\partial y}\right] \\ =\rho\left(u\frac{\partial u}{\partial x}+v\frac{\partial u}{\partial y}+L\Omega^{2}-2\Omega v-\Omega^{2}x\right) \end{matrix}$ (47)

$\begin{matrix} -\frac{\partial}{\partial y}\left(p-\frac{2}{3}\rho k\right)+\frac{\partial}{\partial x}\left[\left(\eta+\eta_{t}\right)\frac{\partial v}{\partial x}\right]+\frac{\partial}{\partial y}\left[\left(\eta+\eta_{t}\right)\frac{\partial v}{\partial y}\right] \\ =\rho\left(u\frac{\partial v}{\partial x}+v\frac{\partial v}{\partial y}+2\Omega u-\Omega^{2}y\right) \end{matrix}$ (48)

When the fluid is incompressible, the standard *k*-*ε* turbulence model is:

$\eta_{t}=C_{\mu}\frac{\rho k^{2}}{\varepsilon}$ (49)

$\rho u\frac{\partial k}{\partial x}+\rho v\frac{\partial k}{\partial y}=\frac{\partial}{\partial x}\left[\left(\eta+\frac{\eta_{t}}{\delta_{k}}\right)\frac{\partial k}{\partial x}\right]+\frac{\partial}{\partial y}\left[\left(\eta+\frac{\eta_{t}}{\delta_{k}}\right)\frac{\partial k}{\partial y}\right]+G_{k}-\rho\varepsilon$ (50)

$\rho u\frac{\partial\varepsilon}{\partial x}+\rho v\frac{\partial\varepsilon}{\partial y}=\frac{\partial}{\partial x}\left[\left(\eta+\frac{\eta_{t}}{\delta_{\varepsilon}}\right)\frac{\partial\varepsilon}{\partial x}\right]+\frac{\partial}{\partial y}\left[\left(\eta+\frac{\eta_{t}}{\delta_{\varepsilon}}\right)\frac{\partial\varepsilon}{\partial y}\right]+\frac{C_{1\varepsilon}\varepsilon}{k}G_{k}-C_{2\varepsilon}\rho\frac{\varepsilon^{2}}{k}$ (51)

*G*_k_ is the production item for *k*. Its formula is:

$G_{k}=\eta_{t}\left\{2\left[\left(\frac{\partial u}{\partial x}\right)^{2}+\left(\frac{\partial v}{\partial y}\right)^{2}\right]+\left(\frac{\partial u}{\partial y}+\frac{\partial v}{\partial x}\right)^{2}+\left(\frac{\partial w}{\partial x}\right)^{2}+\left(\frac{\partial w}{\partial y}\right)^{2}\right\}$ (52)

To facilitate the derivation, the equations can be expressed as an universal equation. If *φ* represents a universal variable (scalar), the general form of differential equations is:

(53)

The governing equations of shear turbulent flow in moving rectangular coordinates are shown in Table 1.

Table 1 The governing equations of shear turbulent flow in the moving rectangular coordinates

|  | *φ* | *Γ* | *R* |
| --- | --- | --- | --- |
| Continuity equation | 1 | 0 | 0 |
| *u* | *u* | *η*_eff_=*η*+*η*_t_ | $-\frac{\partial p_{eff}}{\partial x}-\rho\left(L\Omega^{2}-2\Omega v-\Omega^{2}x\right)$ |
| *v* | *v* | *η*_eff_=*η*+*η*_t_ | $-\frac{\partial p_{eff}}{\partial y}-\rho\left(2\Omega u-\Omega^{2}y\right)$ |
| *w* | *w* | *η*_eff_=*η*+*η*_t_ | $-\rho g-\frac{\partial p}{\partial z}$ |
| *k* | *k* | *η*+*η*_t_ /*δ*_k_ | $G_{k}-\rho\varepsilon$ |
| *ε* | *ε* | *η*+*η*_t_ /*δ_ε_* | $\frac{\varepsilon}{k}\left(C_{1\varepsilon}G_{k}-C_{2\varepsilon}\rho\varepsilon\right)$ |

where,

$p_{eff}=p-\frac{2}{3}\rho k$ (54)

$\delta_{k}=1.0$ (55)

$\delta_{\varepsilon}=1.3$ (56)

(3)The relationship between the polar coordinates and the rectangular coordinates:

$x=C\frac{\text{sinh}\xi}{\cosh\xi-\cos\zeta}$ (57)

$y=C\frac{\text{sin}\zeta}{\cosh\xi-\cos\zeta}$ (58)

where,

$C=\frac{[(R_{\text{i}}^{2}+R_{\text{o}}^{2}-e^{2})^{2}-4R_{\text{i}}^{2}R_{\text{o}}^{2}]^{\frac{1}{2}}}{2e}$ (59)

$R_{i}=-\frac{C}{\sinh\xi_{i}}$ (60)

$R_{o}=-\frac{C}{\sinh\xi_{o}}$ (61)

According to formula (60) and (61),

$\xi_{i}=\mathrm{arcsinh}\left(-\frac{C}{R_{i}}\right)=\ln\left[-\frac{C}{R_{i}}+\sqrt{\left(\frac{C}{R_{i}}\right)^{2}+1}\right]$ (62)

$\xi_{o}=\mathrm{arcsinh}\left(-\frac{C}{R_{o}}\right)=\ln\left[-\frac{C}{R_{o}}+\sqrt{\left(\frac{C}{R_{o}}\right)^{2}+1}\right]$ (63)

When *R*_i_, *R*_o_ and *e* are known, *C*, *ξ*_i_ and *ξ*_o_ can be obtained.

According to the relationship between the polar coordinates and the rectangular coordinates, there are:

$\frac{\partial x}{\partial\xi}=C\frac{\cosh\xi\left(\cosh\xi-\cos\zeta\right)-\text{sinh}\xi\cdot\text{sinh}\xi}{\left(\cosh\xi-\cos\zeta\right)^{2}}=C\frac{1-\cosh\xi\cos\zeta}{\left(\cosh\xi-\cos\zeta\right)^{2}}$ (64)

$\frac{\partial x}{\partial\zeta}=C\frac{-\text{sinh}\xi\cdot\text{sin}\zeta}{\left(\cosh\xi-\cos\zeta\right)^{2}}$ (65)

$\frac{\partial y}{\partial\xi}=C\frac{-\text{sin}\zeta\sinh\xi}{\left(\cosh\xi-\cos\zeta\right)^{2}}$ (66)

$\frac{\partial y}{\partial\zeta}=C\frac{\text{cos}\zeta\left(\cosh\xi-\cos\zeta\right)-\sin\zeta\sin\zeta}{\left(\cosh\xi-\cos\zeta\right)^{2}}=C\frac{\text{cos}\zeta\cosh\xi-1}{\left(\cosh\xi-\cos\zeta\right)^{2}}$ (67)

①The chain guide method

$u\left(x,y\right)$ and $v\left(x,y\right)$ are the velocity on the section of the annular flow in the physical space. The relationship between $\left(x,y\right)$ and $\left(\xi,\zeta\right)$ are $x=x\left(\xi,\zeta\right)$ and $y=y\left(\xi,\zeta\right)$. The relatioship between partial derivatives of *x* and *y* in the physical space and partial derivatives of *ξ* and *ζ* in the computational space is:

$\left|\begin{matrix} \frac{\partial u}{\partial x} \frac{\partial u}{\partial y} \\ \frac{\partial v}{\partial x} \frac{\partial v}{\partial y} \end{matrix}\right|=\left|\begin{matrix} \frac{\partial u}{\partial\xi} \frac{\partial u}{\partial\zeta} \\ \frac{\partial v}{\partial\xi} \frac{\partial v}{\partial\zeta} \end{matrix}\right|\left|\begin{matrix} \frac{\partial\xi}{\partial x} \frac{\partial\zeta}{\partial y} \\ \frac{\partial\zeta}{\partial x} \frac{\partial\zeta}{\partial y} \end{matrix}\right|$ (68)

②The relationship between the derivative of a function and the derivative of its inverse function

There are $x=x\left(\xi,\zeta\right)$ and $y=y\left(\xi,\zeta\right)$, as well as, $\xi=\xi\left(x,y\right)$ and $\zeta=\zeta\left(x,y\right)$, then

$\xi_{x}=\frac{1}{J}y_{\zeta},\zeta_{x}=-\frac{1}{J}y_{\xi},\xi_{y}=-\frac{1}{J}x_{\zeta},\zeta_{y}=\frac{1}{J}x_{\xi}$ (69)

where *J* is the Jacobi factor, defined as:

$J=x_{\xi}y_{\zeta}-x_{\zeta}y_{\xi}$ (70)

Then

$\begin{matrix} J=x_{\xi}y_{\zeta}-x_{\zeta}y_{\xi} \\ =C^{2}\left[\frac{1-\cosh\xi\cos\zeta}{\left(\cosh\xi-\cos\zeta\right)^{2}}\cdot\frac{\text{cos}\zeta\cosh\xi-1}{\left(\cosh\xi-\cos\zeta\right)^{2}}-\frac{-\text{sinh}\xi\cdot\text{sin}\zeta}{\left(\cosh\xi-\cos\zeta\right)^{2}}\cdot\frac{-\text{sin}\zeta\sinh\xi}{\left(\cosh\xi-\cos\zeta\right)^{2}}\right] \\ =-C^{2}\frac{1}{\left(\cosh\xi-\cos\zeta\right)^{2}} \end{matrix}$ (71)

For a function in rectangular coordinates, the derivatives of the universal variable to *x* and *y* are represented as the derivatives of the universal variable to *ξ* and *ζ* by the chain guide method. So for any variable *φ*:

(72)

(73)

The general form of governing equations in moving rectangular coordinates is:

(74)

Each item in the general form of governing equations in moving rectangular coordinates is transformed. The transformation of the convection terms are:

(75)

(76)

Then

(77)

where,

$U=uy_{\zeta}-vx_{\zeta}$ (78)

$V=vx_{\xi}-uy_{\xi}$ (79)

where, *U* and *V* respectively are the velocity components in *ξ* and *ζ* direction in the computational space, called contravariant velocity components.

Correspondingly:

$u=\left(Ux_{\xi}+Vx_{\zeta}\right)/J$ (80)

$v=\left(Uy_{\xi}+Vy_{\zeta}\right)/J$ (81)

Then

(82)

For diffusion terms:

(83)

(84)

Add the formula (83) and (84) together:

(85)

where,

$\alpha=x_{\zeta}^{2}+y_{\zeta}^{2}$ (86)

$\beta=x_{\xi}x_{\zeta}+y_{\xi}y_{\zeta}$ (87)

$\gamma=x_{\xi}^{2}+y_{\xi}^{2}$ (88)

Thus, the general form of governing equations of shear turbulent flow in moving polar coordinates is:

(89)

The source terms in the bipolar coordinates are completely transformed from those in the rectangular coordinates. According to the relationship between bipolar coordinates and rectangular coordinates, convert the source terms on the right side of formula (53) to bipolar coordinates. Then the governing equations of shear turbulent flow in moving polar coordinates are:

$\frac{\partial U}{\partial\xi}+\frac{\partial V}{\partial\zeta}=0$ (90)

(91)

(92)

$\frac{\rho}{J}\left[\frac{\partial\left(Uw\right)}{\partial\xi}+\frac{\partial\left(Vw\right)}{\partial\zeta}\right]=\frac{1}{J}\frac{\partial}{\partial\xi}\left[\frac{\eta_{eff}}{J}\left(\alpha w_{\xi}-\beta w_{\zeta}\right)\right]+\frac{1}{J}\frac{\partial}{\partial\zeta}\left[\frac{\eta_{eff}}{J}\left(-\beta w_{\xi}+\gamma w_{\zeta}\right)\right]-\rho g-\frac{\partial p}{\partial z}$ (93)

$$\begin{matrix} \frac{\rho}{J}\left[\frac{\partial\left(Uk\right)}{\partial\xi}+\frac{\partial\left(Vk\right)}{\partial\zeta}\right]=\frac{1}{J}\frac{\partial}{\partial\xi}\left[\frac{\eta_{eff}}{J}\left(\alpha k_{\xi}-\beta k_{\zeta}\right)\right]+\frac{1}{J}\frac{\partial}{\partial\zeta}\left[\frac{\eta_{eff}}{J}\left(-\beta k_{\xi}+\gamma k_{\zeta}\right)\right] \\ +\eta_{t}\left\{2\left[\left(\frac{\partial u}{\partial\xi}\frac{y_{\zeta}}{J}-\frac{\partial u}{\partial\zeta}\frac{y_{\xi}}{J}\right)^{2}+\left(-\frac{\partial v}{\partial\xi}\frac{x_{\zeta}}{J}+\frac{\partial v}{\partial\zeta}\frac{x_{\xi}}{J}\right)^{2}\right]+\left(-\frac{\partial u}{\partial\xi}\frac{x_{\zeta}}{J}+\frac{\partial u}{\partial\zeta}\frac{x_{\xi}}{J}+\frac{\partial v}{\partial\xi}\frac{y_{\zeta}}{J}-\frac{\partial v}{\partial\zeta}\frac{y_{\xi}}{J}\right)^{2}\right. \\ \left.+\left(\frac{\partial w}{\partial\xi}\frac{y_{\zeta}}{J}-\frac{\partial w}{\partial\zeta}\frac{y_{\xi}}{J}\right)^{2}+\left(-\frac{\partial w}{\partial\xi}\frac{x_{\zeta}}{J}+\frac{\partial w}{\partial\zeta}\frac{x_{\xi}}{J}\right)^{2}\right\}-\rho\varepsilon\end{matrix}$$

(94)

$$\begin{matrix} \frac{\rho}{J}\left[\frac{\partial\left(U\varepsilon\right)}{\partial\xi}+\frac{\partial\left(V\varepsilon\right)}{\partial\zeta}\right]=\frac{1}{J}\frac{\partial}{\partial\xi}\left[\frac{\eta_{eff}}{J}\left(\alpha\varepsilon_{\xi}-\beta\varepsilon_{\zeta}\right)\right]+\frac{1}{J}\frac{\partial}{\partial\zeta}\left[\frac{\eta_{eff}}{J}\left(-\beta\varepsilon_{\xi}+\gamma\varepsilon_{\zeta}\right)\right] \\ +\frac{\varepsilon}{k}\left\{C_{1\varepsilon}\eta_{t}\left\{2\left[\left(\frac{\partial u}{\partial\xi}\frac{y_{\zeta}}{J}-\frac{\partial u}{\partial\zeta}\frac{y_{\xi}}{J}\right)^{2}+\left(-\frac{\partial v}{\partial\xi}\frac{x_{\zeta}}{J}+\frac{\partial v}{\partial\zeta}\frac{x_{\xi}}{J}\right)^{2}\right]\right.\right.+\left(-\frac{\partial u}{\partial\xi}\frac{x_{\zeta}}{J}+\frac{\partial u}{\partial\zeta}\frac{x_{\xi}}{J}+\frac{\partial v}{\partial\xi}\frac{y_{\zeta}}{J}-\frac{\partial v}{\partial\zeta}\frac{y_{\xi}}{J}\right)^{2} \\ \left.\left.+\left(\frac{\partial w}{\partial\xi}\frac{y_{\zeta}}{J}-\frac{\partial w}{\partial\zeta}\frac{y_{\xi}}{J}\right)^{2}+\left(-\frac{\partial w}{\partial\xi}\frac{x_{\zeta}}{J}+\frac{\partial w}{\partial\zeta}\frac{x_{\xi}}{J}\right)^{2}\right\}-C_{2\varepsilon}\rho\varepsilon\right\} \end{matrix}$$

(95)

where,

$\eta=\eta\left(I_{2}\right)=k\left(I_{2}\right)^{n-1}$ (96)

(97)
